# Supplementary material for: Whole Exome Sequencing to Find Candidate Variants for the Prediction of Kidney Transplantation Efficacy
Source: Genes (Basel). 2023 Jun 11;14(6):1251. doi: 10.3390/genes14061251 (PMC10298443; doi:10.3390/genes14061251)
Supplement: Supplementary file 1 [file genes-14-01251-s001.zip › Submission_statement_EVA (1).pdf]

European Variation Archive
c/o European Bioinformatics Institute
Wellcome Trust Genome Campus
Hinxton
Cambridge
CB10 1SD
United Kingdom 2022.05.4

To whom it may concern,

This document refers to submission account # **885754** (frontiers in genetics Journal), which will be used to submit data and metadata to the European Variation Archive (EVA).

Please be advised that <**Seyed Mohammad Kazem Aghamir (mkaghamir@tums.ac.ir)**> is authorized to upload data to the EVA for archiving and distribution as part of your submission process.

I confirm that we have obtained subject consent that allows the deposit of individual genotype data for <*Whole Exome Sequencing to Find Candidate Variants for the Prediction of Kidney Transplantation Efficacy* > in a public repository.

We understand that should any information referenced in this document be subject to change, an updated Submission statements document should be provided to the EVA.

Sincerely,

<Seyed Mohammad Kazem Aghamir and Fatemeh Khatami>

SMK Aghamir

Fatemeh Khatami
